# RSV-induced changes in a 3-dimensional organoid model of human fetal lungs

**DOI:** 10.1371/journal.pone.0265094

**Published:** 2022-03-09

**Authors:** Terri J. Harford, Fariba Rezaee, Briana R. Dye, Jia Fan, Jason R. Spence, Giovanni Piedimonte

**Affiliations:** 1 Department of Cardiovascular & Metabolic Sciences, Lerner Research Institute, Cleveland Clinic Foundation, Cleveland, Ohio, United States of America; 2 Center for Pediatric Pulmonary Medicine, Cleveland Clinic Children’s, Department of Inflammation & Immunity, Lerner Research Institute, Cleveland Clinic Foundation, Cleveland, Ohio, United States of America; 3 Departments of Internal Medicine and Cell & Developmental Biology, University of Michigan, Ann Arbor, Michigan, United States of America; 4 Department of Biochemistry and Molecular Biology, Center for Cellular and Molecular Diagnostics, Tulane University School of Medicine, New Orleans, Louisiana, United States of America; 5 Department of Pediatrics, Tulane University School of Medicine, New Orleans, Louisiana, United States of America; Affiliated Hospital of Jiangsu University, CHINA

## Abstract

We have shown that respiratory syncytial virus (RSV) can spread hematogenously from infected airways of a pregnant woman to the developing fetal lungs *in utero*. This study sought to measure RSV replication, cytopathic effects, and protein expression in human lung organoids (HLOs) reproducing architecture and transcriptional profiles of human fetal lungs during the 1^st^ trimester of gestation. HLOs derived from human pluripotent stem cells were microinjected after 50 or 100 days in culture with medium or recombinant RSV-A2 expressing the red fluorescent protein gene (rrRSV). Infection was monitored by fluorescent microscopy and PCR. Immunohistochemistry and proteomic analysis were performed. RSV infected HLOs in a dose- and time-dependent manner. RSV-infected HLOs increased expression of CC10 (Club cells), but had sparse FOXJ1 (ciliated cells). Disruption of F-actin cytoskeleton was consistent with proteomic data showing a significant increase in Rho GTPases proteins. RSV upregulated the transient receptor potential vanilloid 1 (TRPV_1_) channel and, while β2 adrenergic receptor (β2AR) expression was decreased overall, its phosphorylated form increased. Our data suggest that prenatal RSV infection produces profound changes in fetal lungs’ architecture and expression profiles and maybe an essential precursor of chronic airway dysfunction. expression profiles, and possibly be an important precursor of chronic airway dysfunction.

## Introduction

Respiratory syncytial virus (RSV) is the most common cause of lower respiratory tract infection in infants and young children, with nearly every child being infected by 2 years of age during seasonal outbreaks occurring worldwide in winter months [1]. Because of the limited duration and strength of the anamnestic immune response to this virus, RSV infections recur throughout adult life and are frequently associated with upper and lower respiratory tract morbidity, especially in the elderly and those with underlying lung comorbidities [1].

We have previously shown in animal models transplacental transmission of RSV from the lungs of pregnant rats to the developing lungs of their offspring [2]. More recent studies have confirmed the existence of vertical RSV transmission in humans by: (a) real-time PCR detection of RSV RNA in peripheral blood at birth [3]; (b) RSV replication in human placenta, where it can be accumulated and then transferred to fetal lung tissues by migrating Hofbauer cells [4]; and (c) anti-RSV IgA and IgM in the cord blood of newborns delivered from mothers with a history of upper respiratory tract illness in the third trimester [5]. Also, an independent study confirmed RSV RNA detection in mononuclear cells by droplet digital (dd)PCR in 58% of human cord blood samples tested, with a positivity rate increased up to 80% during winter months [6].

We have also shown that rats born to RSV-infected mothers have reduced TH1 antiviral immunity, amplified neurotrophic signaling leading to airway hyperreactivity, and increased contractility of their lower airways smooth muscle during postnatal reinfections with the same virus [7]. Importantly, these changes persist after secondary reinfections and might provide a plausible explanation for the development of chronic airway dysfunction in a subpopulation of children and adolescents with a history of RSV infections in infancy [8].

However, the structural and functional consequences of a prenatal RSV infection on human fetal lungs are still largely unknown. To this end, we used a 3-dimensional (3D) human lung organoid (HLO) model system derived from step-wise differentiation of human pluripotent stem cells (hPSCs) with molecular and structural features found in replicating human fetal lungs. In stark contrast to 2D models, these HLOs reproduce the diverse cellularity and complex spatial morphology of *in vivo* fetal lungs to allow biologically relevant cell-cell and cell-matrix interactions. Also, RNA sequencing has shown that after 100 days in Matrigel culture, these HLOs are remarkably similar to human fetal lungs at the 2^nd^ trimester of gestation based on global transcriptional profiles, and thus keep pace with human development *in utero*, providing a unique model of lung differentiation and maturation [9].

We infected the HLOs with rrRSV by using a microinjector system to inoculate the virus directly into the lumen of the organoid and then monitored time- and dose-dependency of viral replication. Comparing RSV-infected and non-infected HLOs, we studied cytopathic effects in the principal structural cell types, assessed the expression of key receptors modulating airway function, and ran an extensive proteomic analysis of the supernatants. The results of this study, performed in a physiologically relevant context, provide new insight into the structural and functional anomalies caused by prenatal RSV infection in developing human lungs.

## Results

**Fig 1:**
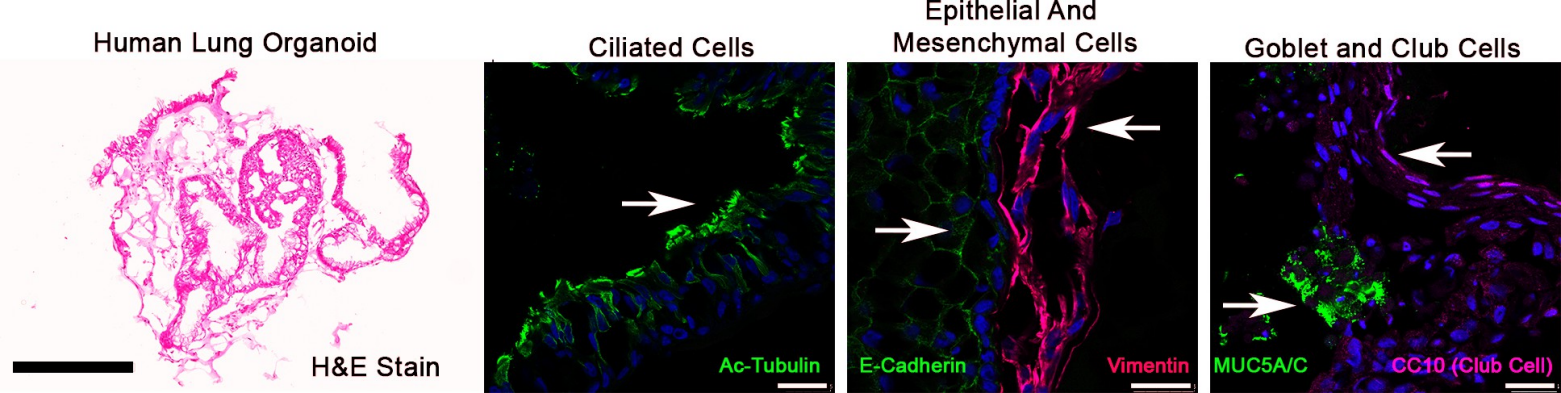
Human lung organoids (HLOs). The HLOs used in this study replicated the 3-dimensional architecture and cell composition of human fetal lungs such as ciliated cell, mesenchymal cells, goblet and club cells. The HLO shown in the left panel was 50 days old. Scale bar left panel 300μm, scale bars in the right three panels are 25μm.

Previous data have shown that HLOs grown under the same conditions described herein were assessed for transcriptional activity by publicly available RNAseq datasets compared to human fetal lungs representing a range of gestational stages. These data showed the HLOs transcriptional activity shares the greatest degree of similarity to human fetal lung and recapitulates gestational age corresponding to the time spent in culture [9]. Here, we show that these HLOs using the protocols outlined in the Method section replicated the 3D architecture and cell composition of human lungs, including ciliated, epithelial, mesenchymal, goblet and Club cells (**Fig 1**).

**Fig 2:**
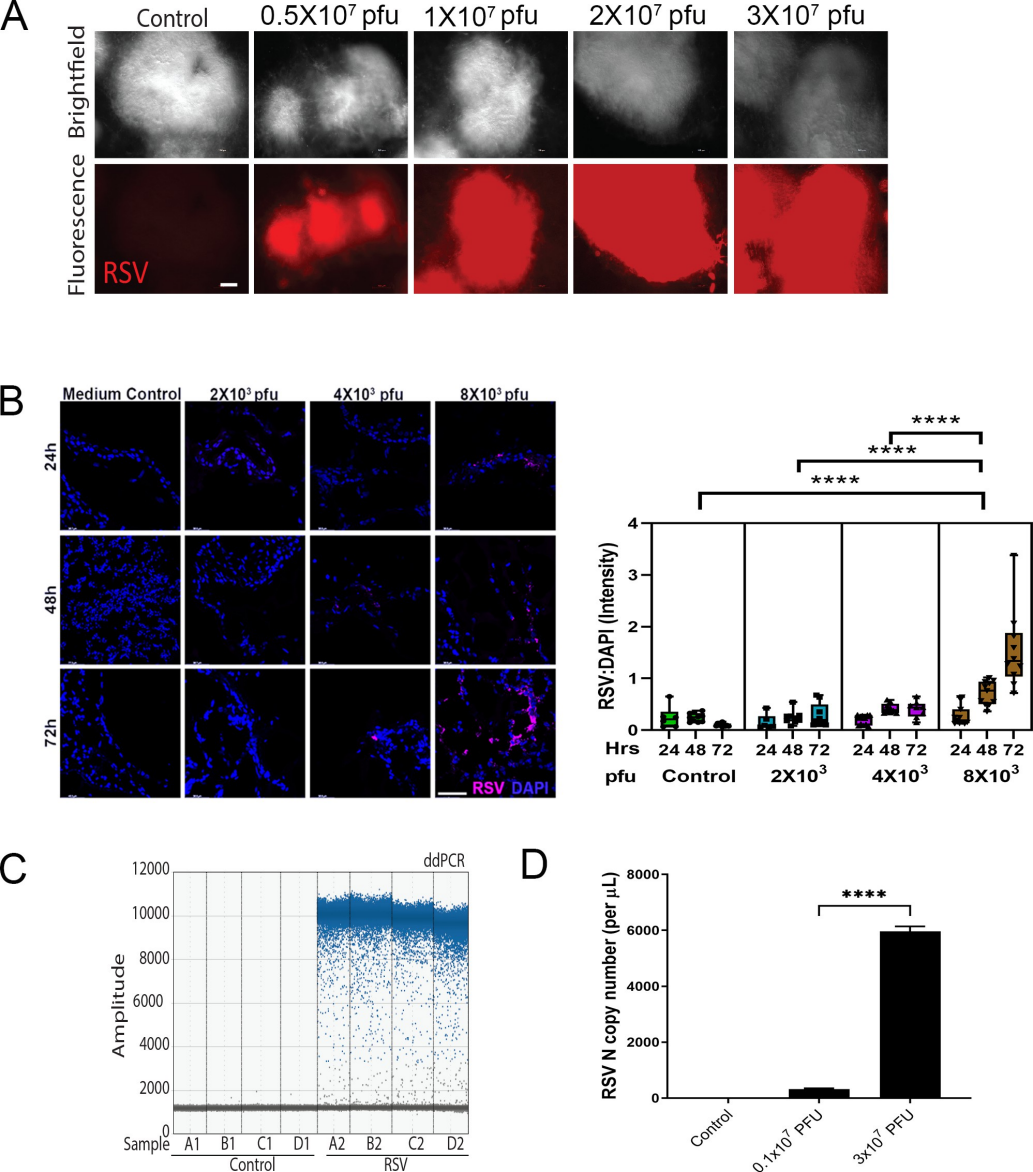
Dose-dependent RSV infection of HLOs. Increasing volumes of rrRSV suspension (10^7^ pfu/ml) or sterile medium were used to infect HLOs using a microinjector system. **(A)** Red fluorescence was expressed 5 days after inoculation by the replication of rrRSV in a dose-dependent manner. **(B)** After inoculation of control sterile medium or increased amounts of rrRSV suspension (10^7^ pfu/ml) into the of HLOs lumen, little infection was noted at 24 h, whereas increasing infectivity was noted at 48 and 72 hours post-infection. **(C)** ddPCR detection of viral RNA in culture medium. **(D)** copy number of RSV nucleocapsid (N) genes in culture medium, confirming a dose-dependent infection.

To determine if RSV infection occurs in a dose-dependent manner, we inoculated the HLOs embedded in matrigel with either sterile medium or rrRSV suspension (10^7^ pfu/ml) by adding RSV infection apically to medium. After 5 days, we could see by fluorescent microscopy evidence of RSV infection in a dose-dependent manner (**Fig 2A**). Subsequently, we inoculated the lumen of the HLOs by adding RSV utilizing the microinjector system described in the Methods section. Immunofluorescence staining of infected HLOs showed a dose- and time-dependent infection, with minimal immunoreactivity visible at 24 hours and progressively increased infectivity noted at 48 and 72 hours post-infection after inoculation (**Fig 2B**). We also detected the viral RNA using ddPCR (**Fig 2C**) and measured the copy number of RSV nucleocapsid (N) protein transcripts to confirm the dose-dependency of the infection (**Fig 2D**).

**Fig 3:**
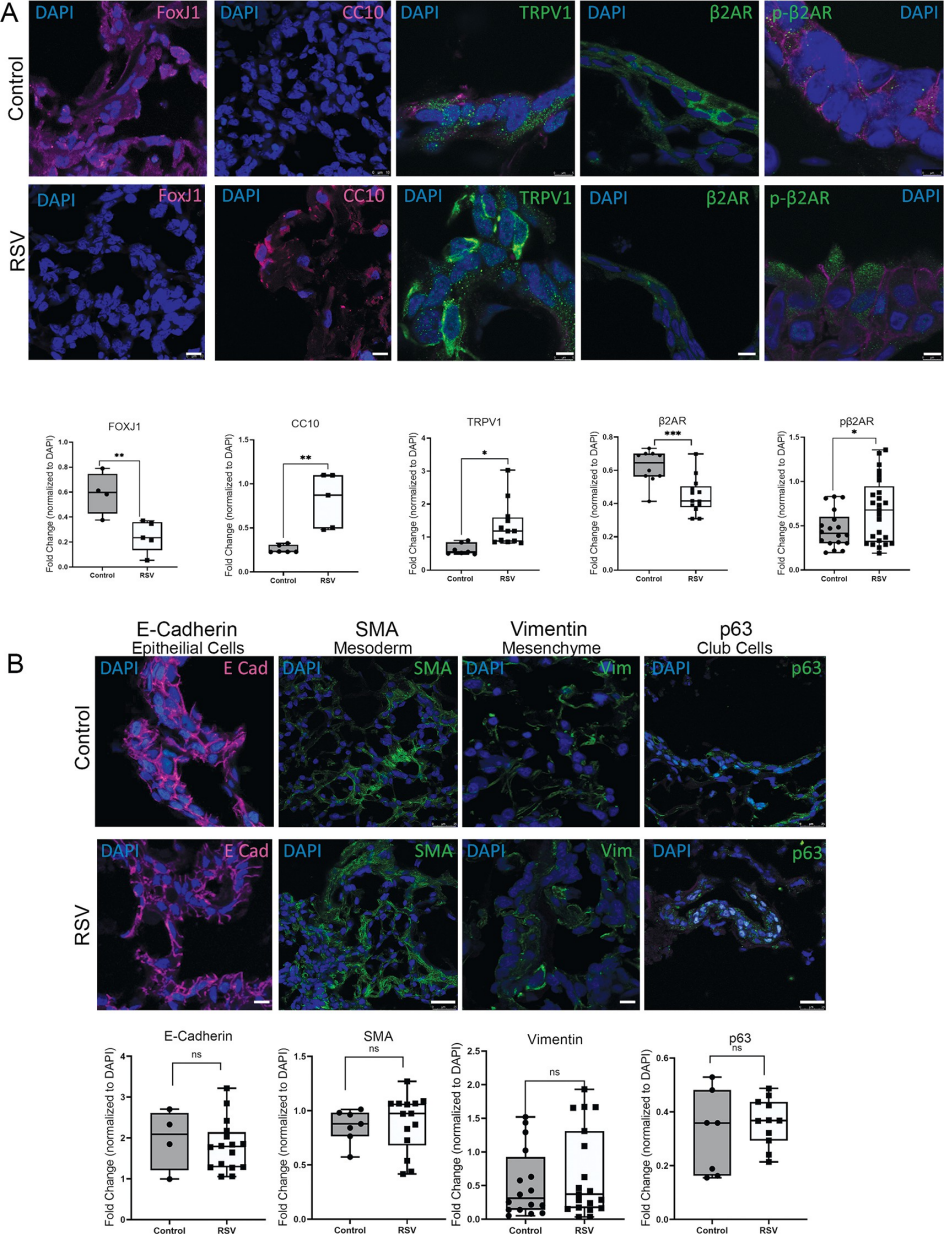
RSV effect on key protein expression. **(A)** RSV infection increased expression of the Club cells’ marker CC10, as well as the transient receptor potential vanilloid 1 (TRPV_1_) calcium channel in mesenchymal cells. In contrast, the expression of ciliagenesis marker FoxJ1 was inhibited. While the expression of β2ARs was reduced, its phosphorylated fraction increased after RSV infection. **(B)** RSV infection did not affect the total expression of the epithelial cells’ marker E-cadherin, smooth muscle cells’ marker actin, mesenchymal cells’ marker vimentin, and basal cells’ marker p63. Graphs below are densitometry of images of a minimum 5 organoids for each control or RSV for each stain.

**Fig 4:**
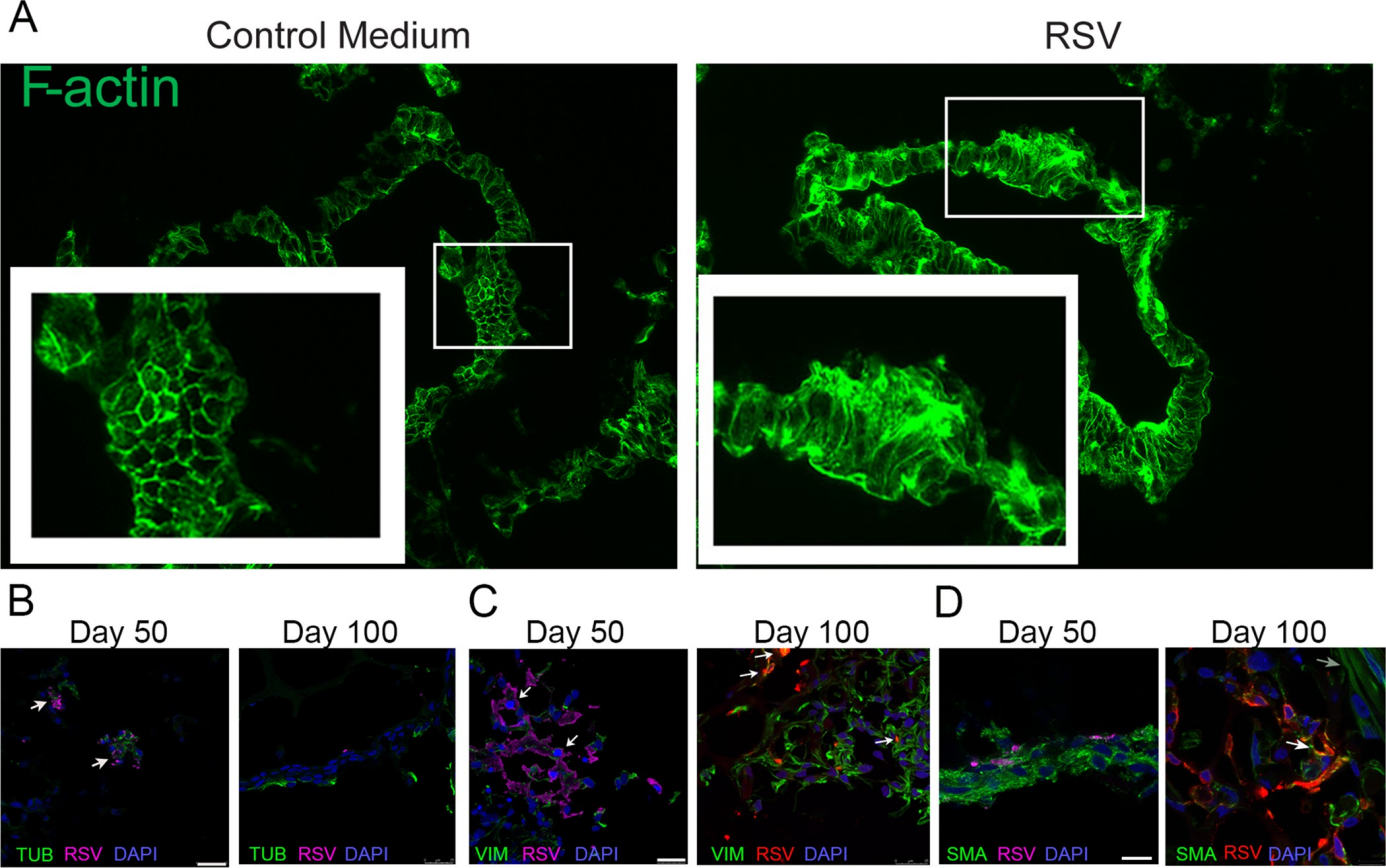
RSV-induced Remodeling of cytoskeletal proteins. **(A)** At 5 days after RSV infection, immunohistochemical staining for F-actin shows extensive remodeling and rearrangement of the HLO’s cytoskeletal architecture. This pattern is consistent with the role played by actin and profilin in virus-mediated cell fusion and viral maturation. HLOs at 50 and 100 days in culture, infected with 8X10^3^ pfu rrRSV for 72 hours, and immunohistochemical staining for **(B)** Tubulin and RSV **(C)** vimentin and RSV and **(D)** smooth muscle actin and RSV shows viral infection is greater in 50 days HLOs. Scale bars 25μm.

Furthermore, in HLOs 50 days in culture, RSV infection led to an increased expression of the Club cells’ marker CC10, as well as the transient receptor potential vanilloid 1 (TRPV_1_) calcium channel in mesenchymal cells (**Fig 3A**). In contrast, expression of the ciliated cells’ marker FoxJ1 was reduced and, while the overall expression of total β2 adrenergic receptors (β2AR) decreased in E-cadherin-positive epithelial cells, its phosphorylated form increased. RSV infection did not affect the expression of the epithelial cells’ marker E-cadherin, smooth muscle cells’ marker actin, mesenchymal cells’ marker vimentin, and basal cells’ marker p63 (**Fig 3B**). However, RSV infection disrupted the E-cadherin structure. (**Fig 4A**). In order to better understand which cell population is susceptible to RSV infection, HLOs in culture for either 50 or 100 days were infected with rrRSV for 72 hours then immunocytochemistry was performed by costaining for both RSV and markers of each cell type. HLOs in culture for 50 days showed tubulin-positive, SMA-positive and vimentin-positive cells were infected with RSV (**Fig 4B**). HLOs in culture for 100 days showed SMA-positive (**Fig 4C**) and vimentin-positive expressing cells (**Fig 4D**) were infected with rrRSV, however we were unable to detect evidence of RSV infection in tubulin-positive expressing cells. Further, in HLOs in culture for 100 days, SMA appeared more organized towards the periphery of the HLO, and some cells exhibited elongated spindle shape that might have been indicative of mesenchymal cells differentiation into smooth muscle cells or fibroblasts. In addition, we started noticing spontaneous contraction of the HLOs within the Matrigel matrix (**S1 Video**). However, we were unable to determine whether the frequency and intensity of the contractions were affected by RSV infection.

**Fig 5:**
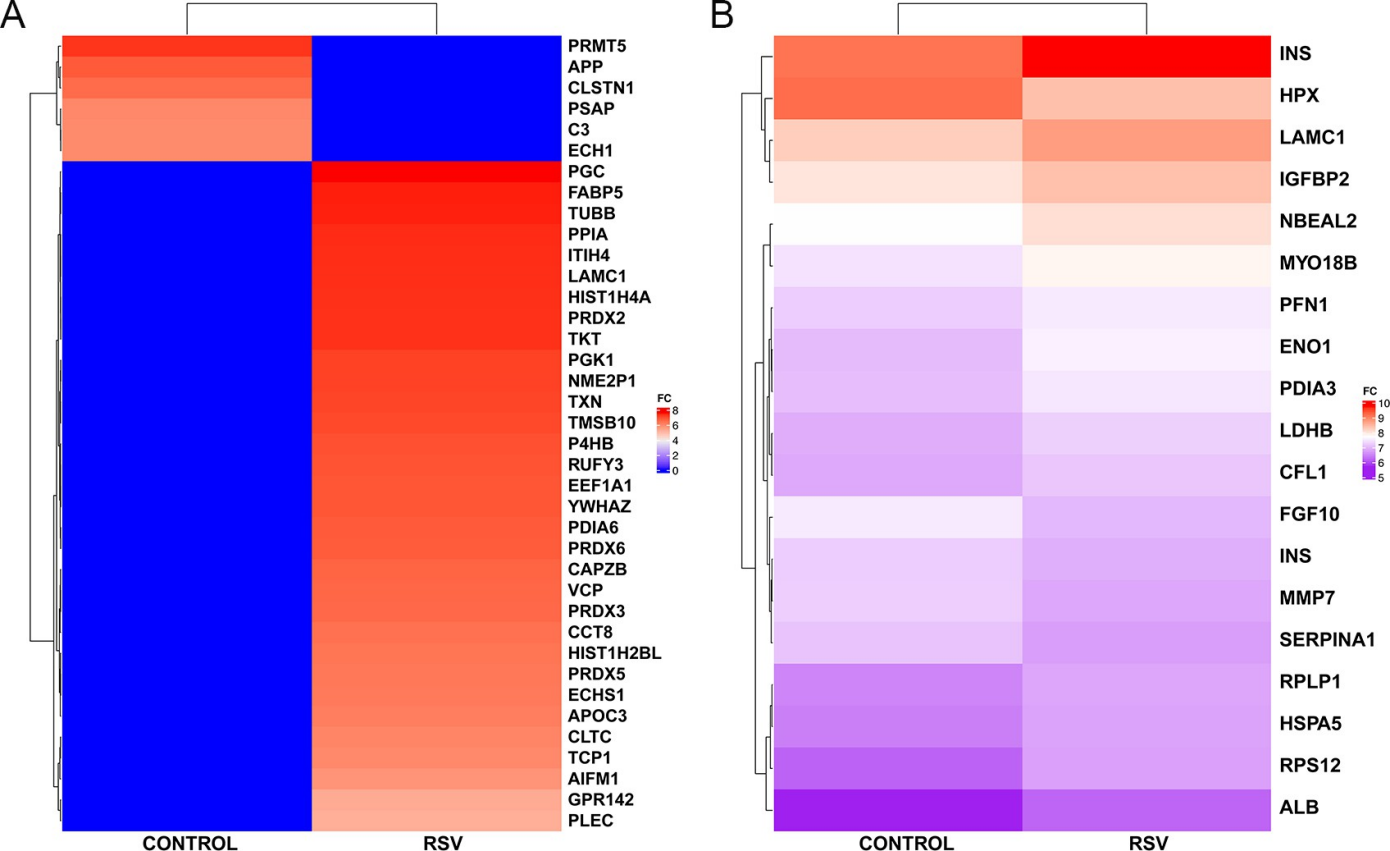
Proteomic analysis of RSV-infected organoids. Heatmaps of dysregulated proteins exhibiting ≥2-fold change in RSV-infected vs. non-infected control organoids, using discrete color to represent proteins abundance after log10-transform. **(A)** Proteins are uniquely identified in either control or RSV groups. Blue label means no signals were being captured for the corresponding proteins. **(B)** Proteins were identified in both control and RSV groups but with at least two-fold changes between groups.

Proteomic analysis identified a total of 95 proteins, and quantitative mass spectrometry was used to analyze differences in protein abundance between RSV-infected vs. non-infected control organoids. After excluding proteins that differed by less than two fold between groups, we selected 55 proteins, including 32 proteins solely identified in the RSV-infected samples and 6 proteins solely detectable in control samples for further analysis (**Fig 5A**). Another 17 proteins were identified in both RSV-infected and non-infected organoids but with different expression levels (**Fig 5B**).

**Fig 6:**
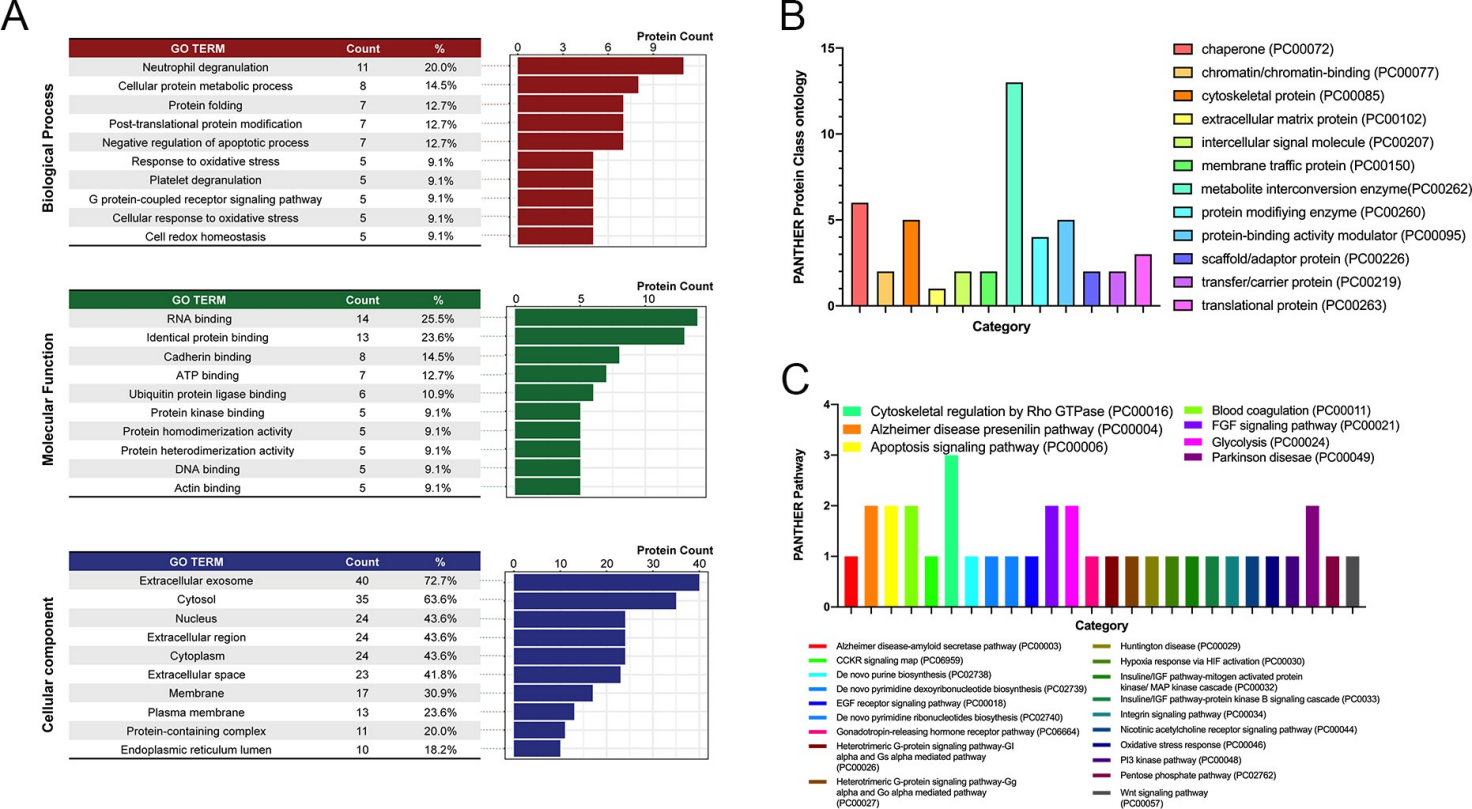
Gene ontology annotation of differentially expressed proteins, and PANTHER pathway analysis. **(A)** The 55 proteins exhibiting significant changes were searched against GO categories from the UniProt database, including biological process, molecular function, and cellular component. Proteins were counted in each ontology they belonged to, and percentages indicate the proportion of the 55 proteins belonging to each class. Bar charts indicate the number of proteins differentially expressed after RSV infection that belong to a specific PANTHER **(B)** Protein Class ontology or **(C)** Pathway. Three proteins (cofilin-1, profilin-1, and tubulin β-chain) were associated with the “Cytoskeletal Regulation by Rho GTPase” pathway. Five other pathways matched two proteins, and 19 pathways matched a single protein.

To further understand these changes in protein expression, we analyzed the 55 uniquely identified proteins against a UniProt Gene Ontology (GO) database and retrieved the top 10 annotations for three GO categorical analyses (**Fig 6A**). The GO terms “Neutrophil Degranulation”, “RNA Binding”, and “Extracellular Exosome” ranked first in the GO Biological Process, GO Molecular Function, and GO Cellular Component categories respectively, non-exclusively accounting for 20%, 26% and 73% of the uniquely identified proteins in each comparison. Of course, the HLOs did not contain neutrophils or other blood cells, but, of the 11 proteins included in the “Neutrophil Degranulation” category, most are involved in viral replication and assembly, cytoskeletal organization, cellular responses to viral invasion (apoptosis and autophagy), and oxidative stress, which is likely to play a fundamental role in the pathogenesis of RSV-induced inflammation and correlates with the severity of clinical illness [10].

Protein classes and pathways that were enriched for these proteins were also assessed by comparison to the PANTHER database (**Fig 6B and 6C**). The most protein hits were detected for the “Metabolite Interconversion Enzyme” class and “Lyase”, “Oxidoreductase”, and “Transferase” sub-classes. A PANTHER pathway analysis found 3 proteins (cofilin-1, profilin-1, and tubulin β-chain) associated with the “Cytoskeletal Regulation by Rho GTPase” pathway, 5 other pathways matched two proteins, and 19 pathways matched a single protein.

## Discussion

This study shows that in a 3D organoid system, which has been previously shown to recapitulate the cytological and transcriptional profile of human fetal lungs during the first trimester of pregnancy, RSV infection occurs in a time- and dose-dependent fashion. Consistent with previous data [11, 12], RSV replicated better in the SMA-positive mesoderm and vimentin-positive mesenchyme—i.e., the airway smooth muscle (ASM) progenitors [13]—than in the ciliated or basal fetal airway epithelium. In contrast, more mature fetal ASM does not support viral replication as efficiently. In mature human [1] and murine [2] airways RSV shows elective tropism for ciliated cells and alveolar epithelial cells. However, in HLOs, the present data suggest that RSV infectivity varies substantially with gestational age and stage of differentiation. In addition, the prenatal ASM susceptibility to RSV may be a precursor of the significant increase in airway reactivity and contractility during early-life RSV infection after *in utero* exposure to the same virus [7].

RSV infection modified the architecture and cytological profile of HLOs by decreasing the population of FoxJ1-positive ciliated cells. The inhibition of ciliagenesis and epithelial metaplasia are known cytopathic effects of this virus also in mature airways and play a central role in the pathogenesis of airway obstruction during acute bronchiolitis and exacerbations of chronic obstructive pulmonary disease (COPD) [14]. RSV infection led to an increase in CC10-positive Club cells that help repair airway damage after infections and produce the Club cell secretory protein (CCSP), known to have anti-inflammatory and immunomodulatory functions in RSV-infected lung [15]. In contrast, no significant change was detected in basal (p63-positive) and non-ciliated (E-cadherin positive) epithelial cell populations after exposure to RSV.

We found that RSV-infected HLOs at 50 and 100 days in culture express higher levels of phosphorylated β2 adrenergic receptors and TRPV_1_ Ca^2+^ channels, respectively in the mesenchymal (E-cadherin-negative) and epithelial (E-cadherin-positive) compartments. Postnatally, pharmacologic agonism of β2ARs typically induces bronchodilation by activating the adenylyl cyclase and generating cyclic adenosine monophosphate (cAMP), which in turn mediates smooth muscle relaxation through protein kinase A (PKA)-dependent mechanisms. However, we have recently shown that RSV infection results in proteolytic cleavage of β2AR, non-canonical activation of adenylyl cyclase impairing cAMP synthesis, and increased muscle contractility due to higher cytosolic calcium concentration, with the net effect of favoring airway obstruction and reducing the bronchodilator effect of β2AR agonists [16].

TRPV_1_ channels expressed in human airways transduce Ca^2+^ in response to airborne chemical and physical irritants. Recent evidence has shown that the lower airways of asthmatic children display elevated basal TRPV_1_ expression and activity, which are further potentiated during RSV infection [17, 18]. Thus, virus-induced rise in intracellular Ca^2+^ mediated by TRPV_1_ activation might contribute to the clinical manifestations of asthma, including augmented synthesis and release of TH_2_ cytokines, mucus overproduction, breakdown in barrier permeability, and increased bronchoconstriction. Taken together, the prenatal effects of acute RSV infection on β2AR and TRPV_1_ expression in fetal lungs could contribute to the predisposition of the developing airways to hyperreactivity and inflammation, thereby increasing the risk characteristics of obstructive diseases like bronchiolitis and asthma after birth.

One of the most concerning changes noted in RSV-infected HLOs was a profound rearrangement of the cytoskeletal architecture in host fetal cells. Indeed, cytoskeletal proteins have been shown to play an important role in the transcription of several negative-strand RNA viral genomes. In particular, profilin and actin are essential for RSV maturation and virus-mediated cell fusion [19], with the latter being also packaged within the mature virions. In turn, the virus leads to partial degradation and destabilization of the action network, which is the primary determinant of cytoskeletal strength, cytokinesis, and cellular architecture, resulting in the diffuse pattern of actin immunostaining seen in the infected organoids [20]. Consistently, proteomic analysis revealed that the pathway with the highest number of proteins differentially expressed after RSV infection was “cytoskeletal regulation by the Rho GTPases,” which have been extensively studied in the context of organization and regulation of cell migration [21–23]. One of these Rho GTPases, RhoA, activates its downstream effector, Rho-associated kinase (ROCK), which is responsible for many cell functions, including remodeling of the extracellular matrix, cell mobility, and actin cytoskeleton organization [24–27]. In another recent study, we have shown that RhoA is upregulated by RSV and plays a critical role in RSV-induced lung damage [28].

RSV-induced changes in the structure and function of the cytoskeleton and ASM are expected to affect the contractile activity of fetal lungs. Interestingly morphological analysis of RSV-infected airway organoids (AO) by another laboratory revealed cytoskeletal rearrangements similar to those shown in our lung model, as well as motility of the organoid in suspension probably generated spontaneous contractions [29]. As recent studies indicate that prenatal ASM regulates lung development via its phasic contractility and growth factor production—which is normal in prenatal life and is governed by a hierarchy of pacemakers within the proximal airways [30]–it is conceivable that interference with the physiologic fetal lung contraction may be an important link between prenatal lung infections and postnatal pulmonary morbidity. Intriguingly, the same pacemakers are one of the putative targets of bronchial thermoplasty therapy for asthma. Future studies using both *in vitro* and *in vivo* models are necessary to further explore and validate this hypothesis, as well as determine whether this mechanism can play a role in the early pathogenesis of chronic airway obstruction.

Proteomic analysis also suggested that viral replication induces an increase in RNA-binding proteins, which ranked first in the GO molecular function analysis [31]. These proteins are primarily found in stress granules and viral inclusion bodies, are known to be induced by RSV infection and play a role in viral replication. Furthermore, 40 of the 55 changed proteins were listed as “extracellular exosome” in the GO cellular component analysis, suggesting that most of them are released through the exosome pathway during RSV infection. These membrane-enclosed small vesicles are involved in the infection process by modulating the host cellular responses, and RNA viruses like RSV attach to Rab11a-positive exosomes to reach the plasma membrane for egress from host cells [32].

To our knowledge, this is the first study based on the infection by RSV of a 3D HLO model system derived from step-wise differentiation of hPSCs and with molecular and structural features replicating human fetal lungs which elucidates specific cell types susceptible to infection [9]. While a previous report had shown that iPSC-derived lung bud organoids (LBOs) containing only mesoderm and pulmonary endoderm could be infected by RSV at day 170 of Matrigel culture [33], very little information was provided concerning the effects of the infection on specific cell types and key receptors. Furthermore, mature club cells, ciliated cells or basal cells were not observed in the LBOs. Subsequently, RSV was used to infect AO composed only of airway epithelial cells (basal, secretory, and ciliated), but not mesenchymal or alveolar cells [29].

Thus, the data reported herein are unique because we infected organoids consisting of epithelial and mesenchymal compartments organized with structural features similar to the native lung, including airway-like epithelium with basal cells and immature ciliated cells surrounded by smooth muscle and myofibroblasts. Moreover, RNA-sequencing has shown that the same HLOs are remarkably similar to human fetal lung based on global transcriptional profiles [9]. While this feature is frequently considered a major shortcoming of organoid-based models of human disease, in our case, it represented a major advantage because our primary goal was to study the effects of prenatal RSV infection on lung development. Another unavoidable limitation of this model is the lack of immune and neural supply, which nevertheless reproduces the conditions found in fetal lungs throughout most of gestation [34].

## Conclusions

In conclusion, the results of this study support the hypothesis that RSV transmitted transplacentally from the mother can differentially infect multiple cell types in the fetal lungs and have significant structural and functional implications for development. RSV infectivity seems to vary with gestational age and differentiation, with selective tropism for mesenchymal/mesodermal elements in early gestation in contrast with the epithelial tropism in late gestation and after birth. The cytological profile of RSV-infected HLOs shows loss of ciliagenesis and metaplasia with reactive Club cells proliferation, combined with diffuse disruption of the actin cytoskeleton. In addition to the complex structural abnormalities, RSV-infected HLOs also display changes in the expression of key receptors predisposing to the development of airway inflammation and hyperreactivity. Thus, based on these new data, we propose that *in utero* exposure of fetal lungs to vertically transmitted RSV can predispose the offspring to develop chronic airway obstruction independently from immunologic or atopic factors.

## Materials and methods

### Maintenance of human embryonal stem cells (hESCs)

hESCs lines H1 (NIH registry #0043) and H9 (NIH registry #0062) were obtained from WiCell Research Institute. Human ES line UM77-2 (NIH registry #0278) was obtained from the University of Michigan. Induced pluripotent stem cells (iPSC) lines 3–5 and 20–1 were generated at Cincinnati Children’s Hospital as described previously [35]. Stem cells were maintained in Matrigel (BD Biosciences, San Jose, CA) in mTeSR1 medium (STEMCELL Technologies, Vancouver, Canada). hESCs were passaged as described previously [36].

### Differentiation of iPSCs into definitive endoderm

iPSCs differentiation was carried out as described previously [37], using a 4-day Activin A (R&D systems, Minneapolis, MN) differentiation protocol. Cells were treated with Activin A (100 ng/ml) for 3 consecutive days in RPMI-1640 media (Life Technologies, Grand Island, NY) with increasing concentrations of 0%, 0.2% and 2% HyClone defined fetal bovine serum (dFBS, Thermo Scientific, West Palm Beach, FL).

### Directed differentiation into anterior foregut spheroids and lung organoids

After differentiation into definitive endoderm, cells were incubated in foregut media with NOG, SB, 500 ng/ml FGF4 (R&D Systems), and 2 μM CHIR99021 (Chiron, Stemgent) for 4–6 days. After 4 days of treatment with growth factors, 3D floating spheroids were noted in the culture. The spheroids were transferred into Matrigel to support 3D growth as previously described [35]. Briefly, spheroids were embedded in a droplet of Matrigel (BD Bioscience) in one well of a 24-well plate, and incubated at room temperature for 10 min. After the Matrigel solidified, foregut media with 1% fetal bovine serum (FBS, Life Technologies) and 500 ng/ml FGF10 were overlaid and replaced every 4 days. Organoids were transferred into new Matrigel droplets every 10–15 days.

### Immunohistochemistry

HLO were isolated from the Matrigel, fixed in 4% PFA 1 hour at 4°C, and washed in PBS before staining with crystal violet to visualize for embedding in Optimal Cutting Temperature (OCT). Samples were sectioned at 5–7 μm, air dried, permeabilized with 3% Triton X-100 for 5 min prior to blocking in 5% donkey serum (Jackson ImmunoResearch Laboratories, West Grove PA). Slides were incubated with primary antibodies (**Table 1**) overnight at 4°C in a humid chamber prior to incubation in fluorescently labelled secondary antibody for 1 hour, and then mounted using Vectashield mounting medium (Vector Labs, Burlingame, CA) containing DAPI to visualize the cell nuclei prior to imaging. Photomicrographs were taken using a confocal microscope (Leica Microsystems, Wetslar, Germany) with a 405-diode laser to excite DAPI and HeNe laser to excite the Alexa Fluor 488, 568 or 633-labeled secondary antibody.

**Table 1:** Primary antibodies used for immunofluorescence experiments.

PRIMARY ANTIBODY	SOURCE	DILUTION	CLONE
**TRPV**_**1**_	Abcam	1:200	Rabbit
**β-2 adrenergic receptor**	Abcam	1:100	Rabbit EPRN707
**E-cadherin**	Life Technologies	1:300	
**SMA**	Abcam	1:100	Clone E184
**MUC5AC**	Invitrogen	1:100	45M1
**FoxJ1**	eBioScience	1:100	2A5
**CC10**	Santa Cruz	1:200	
**β—tubulin IV**	BioGenex	1:200	ONS1A6
**Vimentin**	Cell Signaling	1:5000	D21H3
**RSV**	Meridian Life Science	1:500	Goat
**Rabbit IgG**	Santa Cruz	1:100	
**p63**	Abcam	1:100	

### Microinjection

To infect the HLOs, we used a RSV-A2 strain expressing the red fluorescent protein (RFP) upon replication (rrRSV), whose original stock was kindly provided by Dr. Mark Peeples (Nationwide Children’s Hospital, Columbus, OH) and Dr. Peter Collins (National Institutes of Health, Bethesda, MD). rrRSV was inoculated into the HLO lumen using a microinjector/micromanipulator (Narishige International, USA). This procedure was conducted under sterile conditions in a BSL-2 level cell culture cabinet using a dissecting microscope (Olympus SZ61 microscope equipped with Leica EC3 camera) to visualize the injection field under 1.5X magnification. A micropipette secured to the micromanipulator was loaded with 10 μl of rrRSV suspension (10^7^ pfu/ml in OptiMem) or sterile medium and was guided into the lumen of the HLO to deliver approximately 1-μl total volume until a slight swelling was noted. In the dose response experiments, 2000, 4000 or 8000 pfu’s were used to inoculate the lumen of the HLOs. A minimum of 3 HLOs in each of 3 wells of a 24-well cell culture plate was used for each condition. HLO infection occurred after 50 days or 100 days in culture and were fixed 72 hours post-infection for further immunohistochemical analysis.

### RSV detection and titration

After 4-hr incubation at 37°C, the inoculum was replaced with fresh 3F (advanced DMEM/F12 supplemented with N2 and B27, 10 mM HEPES, 200 mM L-Glutamine, 5000U/ml penicillin-streptomycin, 500 ng/ml FGF10, and 1% fetal bovine serum) medium and the HLOs were visually inspected using a Leica DMi8 microscope equipped with camera and Prior Lumen 200 laser to detect the red fluorescence generated by active RSV replication. Images were taken daily until the HLOs were removed from the Matrigel either 72 hours or 5 days post-infection and transferred to OCT medium to prepare 5-μm sections for immunohistochemistry. The supernatants were collected for RNA extraction and qPCR with primers specific for the nucleocapsid (N) gene to measure RSV copy numbers. HLOs infection was also confirmed by immunofluorescence, electron microscopy (EM), and detection of cytopathic changes. To measure RSV copy number, RNA was extracted from organoid supernatants using the QiaAmp Viral RNA mini-kit. Then, 140 μl of medium eluted in 60 μl of AVE buffer and 5 μl of the final extract was used in a single 20-μl qPCR reaction containing primer and probe mix designed against the RSV N gene (Primerdesign, Chandler’s Ford, UK) and BioRad Universal Probes One-Step qPCR master mix. Control, 0.1-ml RSV, and 3.0-ml RSV samples were each analyzed in four replicates. Thermal cycling conditions were set according to the qPCR primer/probe detection kit manufacturer’s recommendations. Copy numbers of N transcripts were expressed as copies per microliter of medium.

### Proteomic analysis

The supernatants collected from two RSV-infected organoids and two non-infected controls were filtered with Amicon 3K cutoff filters. All samples were dried in a speedvac and reconstituted with 50 μl of 6M urea, 0.1M Tris-HCl, pH 8 buffer. After being reduced with dithiothreitol and alkylated with iodoacetamide, each sample was digested with 1.5 μg sequencing grade trypsin (Promega, Madison, WI) at room temperature overnight. A second aliquot was added the following morning, and digestion continued for 6 hours. The digested samples were desalted with C18 SPE cartridges and dried in a vacuum centrifugal concentrator. Each sample was reconstituted in 30 μl of 1% acetic acid for LC-MS analysis with a ThermoScientific Fusion Lumos mass spectrometry system. The HPLC column was a Dionex 15 cm x 75 μm internal diameter Acclaim PepMap C18, 2 μm, 100 Å reverse-phase capillary chromatography column. Five-μl volumes of the extract were injected, and the peptides eluted from the column with an acetonitrile/0.1% formic acid gradient at a flow rate of 0.3 μl /min were introduced into the source of the mass spectrometer online. The micro-electrospray ion source was operated at 2.5 kV. The digest was analyzed using the data-dependent multitask capability of the instrument, acquiring full-scan mass spectra to determine peptide molecular weights and product ion spectra to determine amino acid sequence in successive instrument scans.

The relative abundance of identified human proteins was determined using the program MaxQuant and fold change was calculated using the formula: (Abundance_RSV_+1)/(Abundance_CONTROL_+1) [38]. Proteins that were uniquely detected or exhibited a ≥2-fold difference between RSV and control samples were selected for visualization and bioinformatic analysis. Heatmaps of the relative abundance after log10 transformation were generated with the ComplexHeatmap v2.2.2 in the R v4.0.3 programming environment [39]. Gene Ontology (GO) analysis and visualization were performed using the UniProtR v2.0.6 package, which was connected to the UniProt Database to retrieve information [40]. Protein class and pathway analyses were performed using the PANTHER (Protein ANnotation Through Evolutionary Relationship) protein classification system. Because any given protein can be classified in more than one ontology group, the number of proteins associated with the GO terms exceeded the total number of proteins under analysis.

### Statistical analysis

All data are expressed as mean ± SEM. Multiple comparisons were performed with the non-parametric Kruskal-Wallace test and corrected for multiple comparisons with the Dunn’s test. All calculations were performed with GraphPad Prism software version 5.0. *P* < 0.05 was considered to be statistically significant.

## Supporting information

S1 Video Demonstrates spontaneous contracting cells located at the periphery of the HLOs observed within the HLOs embedded in the Matrigel matrix after 100 days in culture resembling contractions typically seen in fetal lungs during branching morphogenesis. (MP4)

S1 Dataset Proteomic data. Figure shows raw proteomic data set.
(XLSX)
